# Supplementary material for: ProteinShader: illustrative rendering of macromolecules
Source: BMC Struct Biol. 2009 Mar 30;9:19. doi: 10.1186/1472-6807-9-19 (PMC2672931; doi:10.1186/1472-6807-9-19)
Supplement: Additional file 1 — ProteinShader program without source code. This compressed file contains the complete ProteinShader program including associated libraries, but no source code. A README.txt file gives an overview of the ProteinShader distribution, and the index.html file in the help subdirectory has directions on getting started with the program as well as a set of tutorials. [file 1472-6807-9-19-S1.zip › ProteinShader-beta-0_9_4-binary/help/api/org/proteinshader/structure/package-frame.html]

org.proteinshader.structure (ProteinShader API)


org.proteinshader.structure

Interfaces 
  
*IDTest*

Classes 
  
AminoAcid
  
Atom
  
BetaStrand
  
Bond
  
Category
  
Chain
  
Description
  
Drawable
  
Helix
  
Heterogen
  
Loop
  
Model
  
Record
  
Region
  
Residue
  
Segment
  
Structure
  
Water
